# The Human Genomic Melting Map

**DOI:** 10.1371/journal.pcbi.0030093

**Published:** 2007-05-18

**Authors:** Fang Liu, Eivind Tøstesen, Jostein K Sundet, Tor-Kristian Jenssen, Christoph Bock, Geir Ivar Jerstad, William G Thilly, Eivind Hovig

**Affiliations:** 1 Department of Tumor Biology, Institute for Cancer Research, Rikshospitalet-Radiumhospitalet Medical Center, Oslo, Norway; 2 PubGene AS, Vinderen, Oslo, Norway; 3 Institute of Informatics, University of Oslo, Norway; 4 Max-Planck-Institut für Informatik, Saarbrücken, Germany; 5 Biological Engineering Division, Massachusetts Institute of Technology, Cambridge, Massachusetts, United States of America; 6 Medical Informatics, Institute for Cancer Research, Rikshospitalet-Radiumhospitalet Medical Center, Oslo, Norway; Ghent University, Belgium

## Abstract

In a living cell, the antiparallel double-stranded helix of DNA is a dynamically changing structure. The structure relates to interactions between and within the DNA strands, and the array of other macromolecules that constitutes functional chromatin. It is only through its changing conformations that DNA can organize and structure a large number of cellular functions. In particular, DNA must locally uncoil, or melt, and become single-stranded for DNA replication, repair, recombination, and transcription to occur. It has previously been shown that this melting occurs cooperatively, whereby several base pairs act in concert to generate melting bubbles, and in this way constitute a domain that behaves as a unit with respect to local DNA single-strandedness. We have applied a melting map calculation to the complete human genome, which provides information about the propensities of forming local bubbles determined from the whole sequence, and present a first report on its basic features, the extent of cooperativity, and correlations to various physical and biological features of the human genome. Globally, the melting map covaries very strongly with GC content. Most importantly, however, cooperativity of DNA denaturation causes this correlation to be weaker at resolutions fewer than 500 bps. This is also the resolution level at which most structural and biological processes occur, signifying the importance of the informational content inherent in the genomic melting map. The human DNA melting map may be further explored at http://meltmap.uio.no.

## Author Summary

In a living cell, DNA both is an information carrier and carries out important structural tasks, such as organizing its replication and distributing the chromosomes to the daughter cells. DNA is frequently depicted as an antiparallel double-stranded helix, but DNA may rather be viewed as having a dynamically changing structure. This is because in performing most of these tasks, it is necessary for the DNA helix to become single-stranded locally, or unwound, thereby creating “bubbles” in the double strand, much as what happens when a twisted rope with two strands is untwisted. In the cell, this happens by the aid of the enzymatic machinery, but it may also be observed in experiments when a gradual increase in temperature produces bubbles. Our calculations in producing a melting map are based on temperature changes, but may be viewed as a map of bubble formation tendencies along the genome. In DNA, an opening bubble does not open one base at a time, but rather as a cooperative event, in that several base pairs act in concert to form a bubble, and we use an algorithm that takes this aspect into consideration. We then explore the correlations between the melting map and many known features of the human genome. We also demonstrate the extent of cooperativity, and find that the melting map carries information otherwise not available. Once the melting map is calculated, a number of more detailed studies of relationships to DNA structure and function are made possible, as well as improvements of algorithms for modelling DNA with associated proteins as they occur in the natural cellular environment.

## Introduction

Currently, a community-wide effort is being pursued to understand how the genome itself, in concert with its epigenetic modifications and its organization in the cell nucleus, functions to provide each cell with the functionality and gene regulation that it requires at various levels of organization, such as cell state and tissue specificity. Two important and distinct approaches towards this goal may be found: (1) data-driven statistical learning methods that can provide results in a relatively short time, with little knowledge of the biological mechanisms involved; (2) knowledge-driven physical modeling that can provide a mechanistic understanding of the system in terms of its molecular constituents. However, by nature the latter is a relatively slow and difficult process.

In most of the fifty years following the discovery of DNA structure [1], the base sequence of DNA has been the primary focus, as epitomized by the completion of the human genome [2,3]. Now, with the sequence in hand, efforts have intensified to relate local sequence motifs or physical characteristics to involvement in particular DNA-dependent processes. Prediction algorithms for a number of DNA features based on sequence information have been developed, such as prediction of genes, alternative splicing, and transcription factor binding [4–6], leading to an increasing number of annotations being available for the most widely studied organisms [7].

Studies of chromatin DNA in its nuclear environment have found that chromosomal compartmentalization occurs in the nucleus, and that apparently most of the genes locate in the inner part of the nucleus [8,9]. The importance of chromatin modification for gene expression has been recognized [10]. Still, many of the organizing principles of the structural elements of DNA within the eukaryotic nucleus remain poorly understood.

The interest in the physical organization of DNA may be considered as a reflection of the fact that many complex biological processes require an integrative approach, given the relative shortage of laboratory-based observations. Sequence-oriented annotations of diverse types, physical modeling, and experimental observation of chromatin organization are now providing rich, yet disparate, sources of information on scales ranging from a few base pairs to the whole genome. A systematic integration and statistical exploration of the combined data is believed to provide a more complete picture of the functionality of DNA in its natural context.

In parallel with the determination of the DNA sequence, efforts have been made to model the molecular behavior of DNA. A central issue has been prediction of how the DNA denatures, or melts, to dynamically create local single-stranded regions, to which a number of single-strand binding elements can attach, and thus influence such functions as gene transcription and a number of other DNA-dependent functions.

Algorithms for this aspect of DNA behavior have been built on the foundations of polymer theory and statistical mechanics, in which the pioneering work of Poland and Scheraga [11] established a method to predict the melting profiles of DNA. When these algorithms were applied to the exonic sequences of specific genes, it became possible to develop technology to separate mixed mutant and wild-type sequences, and to observe the quantitative sequence-specific distribution of point mutations in human tissues and large human populations [12–14].

DNA melting algorithms aim at quantitative predictions of in vitro experiments in dilute DNA solutions at specific temperatures, ionic strengths, and denaturing solutes [15]. Few attempts have been made to bring quantitative modeling inside the cell nucleus. A precise model of chromatin DNA would be very complex, accounting for a number of physical structures. Among these would be the generation and response to superhelical stress [16], the histone-based hierarchical folding, the maintenance of topological order, the nucleus wall–inducing confinement, attachment, and molecular crowding, the protein-driven replication and transcription processes, as well as other nonequilibrium effects to be discovered. Due to the lack of such chromatin models, the aim of the present type of investigation is neither a quantitative prediction in vivo nor in vitro. Rather, as pioneered by L. S. Lerman, who applied a DNA melting algorithm to the human beta-globin gene [14], and by G. J. King [17], who applied melting maps in a study of yeast chromosome III, the thought is that the existing melting algorithms, while describing in vitro experiments, may also reflect qualitatively some of the chromatin DNA properties in vivo. Therefore, the primary interests here are the qualitative features, such as propensities for single-strandedness, rather than the quantitative detail. To produce qualitative information that is physically realistic, however, a quantitatively accurate model and algorithm is required.

An important requirement for any melting algorithm to be useful on long genomic sequences is that its computation time grows linearly with sequence length. For decades, the only available linear algorithm was the Poland–Fixman–Freire (PFF) algorithm [18,19]. It calculates the melting properties according to the classical Poland–Scheraga model [11], originating in the 1960s, which considers a base pair to have simply two distinct states, helix and coil. The key element of the Poland–Scheraga model are the loop entropies [11], whose scaling behaviors have been derived from various random walk polymer models that may take into account excluded volume effects, while other effects, such as chain stiffness in smaller loops (<30 bp), are less well-understood [15]. The statistical weight of interior loops given by the loop entropy factor cannot be expressed as a product over base pairs, which intrinsically makes an exact calculation grow quadratically with sequence length, as in the Poland algorithm [19]. However, an approximation of the loop entropy factor was incorporated into the Poland algorithm in 1977 by M. Fixman and J. J. Freire [18], providing the linear but approximative PFF algorithm. Various implementations of the PFF algorithm have been available in the scientific community, among which Lerman's MELT87 implementation was the first widely available code. Only in recent years have other linear algorithms become available [20,21]. In one such algorithm [21], some of us introduced a Forward–Backward method (analogously to the Poland algorithm) for the recursive calculation of partition functions in the Poland–Scheraga model. Another recent algorithm [20] calculates the melting properties according to the Dauxois–Peyrard–Bishop model [22,23]. This model does not explicitly have a loop entropy factor that could slow down the computation. Instead, the energetics relies on each base pair having a continuum of possible states, mimicking a gradually varying geometry of the hydrogen-bonded bases. Calculations involve an integration over these continuous variables that can be algorithmically complex, but a recent approximative discretization method [24] has provided a linear algorithm that may be applicable to whole-genome computation of melting temperature [20]. Any algorithm that is quadratic or slower could be applied in linear time, by using the basic windowing technique of dividing up the sequence into pieces to be calculated individually and merging the results afterward. Such an Alexandrian solution is usually problematic due to the associated errors. For the Poland–Scheraga model, these errors could be kept small by first locating the pieces according to successive helical regions [25]. However, neither the PFF nor the more recent linear melting algorithms rely on a windowing technique for their speed. For those algorithms, windowing cannot provide much further reduction in computation time, but it may reduce the required RAM considerably. For more complex melting algorithms, on the other hand, windowing may be the only choice for analyzing long genomic sequences. One such example is C. J. Benham's SIDD model of superhelical DNA melting [26–28]. This model has distinct helix and coil states as does the Poland–Scheraga model, but rather than focusing on loop entropies and excluded volume, it models the torsional stresses imposed with a fixed linking number. Unwinding transitions can be induced by both increased temperature and negative superhelicity. SIDD calculations with a windowing technique have identified the bubbles or SIDD sites in yeast and various microbial genomes at physiologically reasonable values of the temperature and linking number [29]. The present work, however, applies the original PFF algorithm, which has the speed required for a human genomic calculation.

Earlier studies similar to the present one have investigated genomic profiles of the local GC contents [30], partly because they provide an indirect reflection of DNA's biophysical properties. It would, however, be misleading to consider the melting temperature as simply a function of the local di-nucleotide distribution. This misconception is propagated by web tools such as the WEB-THERMODYN [31], or the EMBOSS' DAN [32], in which melting temperature predictions appropriate for oligonucleotides are applied to a sliding window in longer sequences. The profiles thus obtained are essentially just the compositional profiles in disguise, and should provide similar results in genomic analyses. The problem with GC contents and sliding window approaches is that they fail to exhibit the cooperative physics. Base pair melting temperatures are determined by the organization of the sequence into cooperative domains of tens to hundreds of base pairs with well-defined boundaries. Each domain has its own melting temperature, resulting in a characteristic appearance of a melting map. The location of the domain boundaries partly depends on the long-range effects of the loop entropies, the extent of which, however, has not been fully investigated. The calculation of a melting map is nontrivial, in the sense that the whole sequence must be considered to account for these long-range effects. Although cooperativity features are absent in GC profiles, some correlation is expected between the melting map and the local GC contents. For example, a genome has a mosaic structure organized on many levels, with GC contents varying between isochores, exons, and introns, and in local motifs with abrupt changes in composition. By applying segmentation algorithms to genomic GC profiles [33,34], a set of boundaries between regions of different GC contents can be found. The GC variations may in some cases “force” the thermodynamic domain boundaries to coincide with the GC-based boundaries, but the a priori expectation is that the two sets of boundary locations are different due to cooperativity. The first step in exploring cooperativity on a genomic level presented here opens a route to unravel novel mechanistic implications of the DNA sequence.

Combining a knowledge-driven modeling approach and data-driven statistical explorations, we now report the complete calculation of the human genomic melting map, and we report a first explorative examination of the potential information content of the melting map. We discuss the large-scale computational challenges, such as the algorithmic complexity, the high-precision floating point formats, a Fixman–Freire approximation for very large sequence lengths, and the hardware requirements. We find that the cooperativity of DNA dominates at sequence resolution below 500 base pairs, and most importantly, that neighboring melting domains influence each other such that GC content is no longer a sufficient predictor of single-strandedness. This has important implications for understanding the interactions in chromatin.

## Methods

### Melting Algorithm

The melting profiles were calculated using the Poland algorithm [19] with the Fixman–Freire approximation [18]. We employed a Fortran implementation that has its origin in the MELT87 program written by L. S. Lerman [14], but we have made important modifications of the code to enable large-scale genomic computations. First, we use data types with ultra-high precision (exponent terms to 4,500, with up to 800 significant digits), to prevent the well-known problem of arithmetic overflow in dynamic programming. Second, we extend the accuracy of the Fixman–Freire approximation to very large loop sizes. In the Fixman–Freire scheme, the loop entropy factor Ω(*x*) = *σ*(2*x* + *d*)^−*α*^ (which is a function of *x*, the number of melted base pairs in a loop plus one) is approximated by Ω′(*x*) = const × *σ* · *f*(2*x* + d), where the power function has been replaced by some multi-exponential function

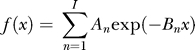
The parameters *I*, *A_n_,* and *B_n_* (*n* = 1,..,*I*) can be determined by fitting *f*(*x*) to *x* ^−*α*^ *.* The MELT87 code contained a hard-coded set of *I* = 10 exponentials. Although it is known that with a fixed number *I,* the Fixman–Freire approximation breaks down for long enough sequences, the consequences for the melting calculations have been largely ignored in the literature. Figure 1 shows a plot of the exact loop entropy factor Ω(x), together with an *I* = 10 and an *I* = 21 approximation. The *I* = 10 approximation is only accurate for loop sizes up to the order of 10^4^, whereafter it decreases exponentially. The *I* = 21 approximation is accurate for loop sizes up to the order of 10^8^, whereafter it also decreases exponentially. When we first applied an *I* = 10 approximation in the calculation for human chromosomes, we observed “ceiling” artefacts imposing upper limits on the melting temperatures. An interpretation of this observation is that very large loops should be included in the partition function at high temperatures, despite their low statistical weights. To take large loops properly into account, we derived a Fixman–Freire approximation for arbitrary sequence length. Here, the parameters *I*, *A_n_,* and *B_n_* are given algebraically by the following expressions: *I* ≥ 1 + ln(2N) where N is the sequence length, *B_n_* = *e^n−I^*, and

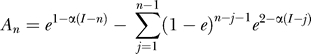
Previously, such parameters have been obtained using more complex algorithms for solving the curve-fitting problem. The straightforward mathematical expressions provided here may be advantageous in terms of simpler programming and numerical reproducibility. We have set up a Web tool for calculating these parameters for given user input, including graphs that show the accuracy of the approximation (see http://meltmap.uio.no/tools/loopentropy.html).

**Figure 1:**
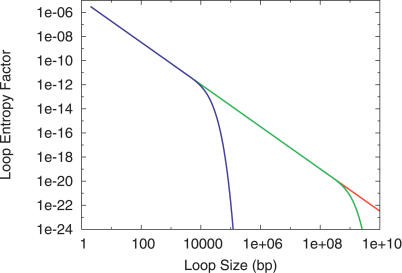
Loop Entropy Factor Estimation The exact loop entropy factor for *σ* = 3.5 · 10^−5^, *α* = 1.75, and *d* = 0 is plotted (red) as a function of loop size, together with two Fixman–Freire approximations: a 10-exponentials approximation (blue), which is valid up to loop size about 10^4^, and a 21-exponentials approximation (green), which is valid up to loop size about 10^8^.

For all the human chromosomes, we used the set of *I* = 21 exponentials. The extra computation time spent (about twice that for an *I* = 10 set) removes the artefacts that would otherwise have required extra validation efforts.

The Poland algorithm also uses parameters that determine the free energy contributions of the helical segments. Several sets of experimentally determined parameters are available, but a comparative study by SantaLucia [35] has shown that a consensus among them exists. The choice of parameters should not be very important for the present type of study. Here we use Gotoh and Tagashira's ten nearest neighbor parameters [36]. An advantage of this set is that it was specifically designed for the Poland algorithm, with their modification that free energies are assigned to nearest-neighbor doublets rather than base pairs, and the parameters were determined by fitting calculated melting curves to experimental curves (at salt concentration 19.5 mM). For the loop entropy factor, we used *σ* = 3.5 · 10^−5^, *α* = 1.75, and *d* = 0. We do not distinguish methylated cytosine from unmethylated cytosine, and we use the parameters for unmethylated cytosine in both cases. Unknown bases in the sequence (denoted by N) are assigned their own parameters obtained by averaging over the four bases A, C, G, and T.

The output of the Poland algorithm is a probability profile showing the probability of each position to be in the helical state calculated for a given temperature. For each chromosome, we calculate all probability profiles in the range 45° C to 110 °C at every 0.1 °C temperature increment. From this set of probability profiles, we derive the melting temperatures Tm(*x*) at which the probability at position *x* equals 50%. The resulting Tm-profiles or melting maps summarize the main features of melting along the sequences. The melting maps are stored in a format rounded to two digits after the decimal point. The complete calculation of probability profiles for all human chromosomes takes approximately 22 CPU days on an HP Superdome (64 × Itanium 2 processors, 1.5 Ghz, 6 MB cache). The calculation requires at least 13 GB RAM to process the longest chromosome (~240 Mbps) with seven arrays extended precision.

In some of the downstream analyses, we used “zoomed-out” melting maps, i.e., averaging melting temperatures in nonoverlapping windows of a certain size (varied from 10 bp to 1 Mbp). As the melting maps represent cooperative melting events of many base pairs, many features are still present at a lower resolution.

### Source Sequence

UCSC Golden Path Human Genome Sequence Release *hg17* (May 2004) [37], containing the *Build35* assembled by the International Human Genome Project sequencing centers, were downloaded and used in our calculation of DNA melting profiles.

### Randomized Chromosomes

We also calculated another set of melting profiles for 24 randomized chromosome sequences. When generating the randomized chromosomes, we ensured that the total length, and the number of A, T, G, C, and N of each chromosome (N represents unknown bases, which are mostly located in euchromatic gaps), as well as the start and end positions of each consecutive N stretch, correspond to their human chromosome counterparts. Only the base compositions, not the di- or tri-nucleotide compositions, were specified. The randomized chromosomes are not completely featureless, however, since they contain the same stretches of N's as in the human chromosomes. The melting map algorithm was executed with the randomized chromosome set as input, and all the downstream statistical analyses were performed similarly.

### Melting Domain Segmentations

A characteristic of a melting domain is that each base pair has the same melting temperature. The flat plateaus of a melting map may give an indication of the location of domains. Two alternative segmentation methods were developed to identify flat segments of the melting map.

First, we identified *flat* and *nonflat* segments of a given constant size (e.g*.*, 100 bp or 1 Kbp) by ranking the standard deviations (SDs) of the melting temperatures within nonoverlapping windows. Those segments having high SDs were designated as *nonflat,* and the ones with low SDs were designated as *flat*.

Second, we also defined three types of segments, called *up, down,* and *flat,* based on the stepwise change in melting temperature, ΔTm = Tm(*x* + 1) − Tm(*x*), between neighboring positions within a segment (in the 5′ to 3′ direction). An *up* segment was defined as a consecutive series of stepwise increases in Tm by at least ΔTm ≥ 0.13°C. Vice versa, a *down* segment was defined as a consecutive series of stepwise decreases by ΔTm ≤ −0.13°C. The *flat* segments consisted of small stepwise changes of |ΔTm | ≤ 0.01 °C. Neighboring positions with intermediate-sized stepwise change (0.01 °C < |ΔTm | < 0.13 °C) were not assigned to any segment. In Chromosome 21, 1.07% of all neighboring positions were part of *up* segments, 1.07% were part of *down* segments, 88.82% were part of *flat* segments, and the rest was not categorized. For each identified segment [*x*_start_, *x*_end_], the length L = *x*_end_ − *x*_start_ + 1, the average melting temperature, and the end-to-end step height (Tm(*x*_end_) − Tm(*x*_start_)), were determined. Segments of L < 9 were discarded for downstream analyses.

### Statistical Association between the Melting Map and DNA Sequence Properties

The Pearson correlation coefficient was used to quantify the direction and magnitude of coordinated relations between various pairs of continuous variables, for example, the melting temperature versus the GC contents, and the recombination rates, respectively.

The local GC content was calculated for every nonoverlapping window of different lengths (varied from 10 bps to 1 Mbps) as the ratio of G + C over the total number of A + T + G + C within each window. Note that the Ns do not contribute in the above definition, i.e., we made no assumptions of the base pair composition with respect to unknown bases.

When investigating the correlation between melting profiles and the recombination rates, we used the DeCODE data source, as publicly available [38,39]. This set of data has a resolution of 1 Mbp.

For exploratory analysis of annotation correlations, the EpiGRAPH analysis service [40,41] was utilized. This service provided statistical association analysis for more than 1,000 human genomic annotations, including DNA sequence properties and patterns, repeat frequency and distribution, CpG island frequency and distribution, predicted DNA structure properties, predicted transcription binding sites, evolutionary conservation, and SNPs. These annotations were tested against discretized pairwise classes of input melting regions. As these calculations were computationally demanding, only sets of up to 100 cases per class were performed for each analysis.

### Spectral Analysis

The enormous size of the human genome, with the shortest chromosome being more than 45 Mbps, requires an approach that can, beyond local details, reveal possible global patterns in an analysis of the melting map. Wavelet analysis, having evolved from Fourier transformations, has become an increasingly popular and useful tool for analyzing signals that contain nonstationary power at many different frequencies. It has been found in previous studies [42,43] using wavelet analysis of the GC contents of human chromosome sequences that regular nonlinear oscillatory behavior occurs. By association to the same underlying organizational principles, we therefore similarly examined if we could identify wavelengths by spectral analysis of the calculated DNA melting map. We applied the Morlet wavelet [44] to identify possible dominant periodic components in the melting map, using MATLAB Wavelet Toolbox (The MathWorks, http://www.mathworks.com). Although the wavelet decomposition algorithm has been under continuous improvement since its inception, wavelet analysis is still computationally demanding.

We performed continuous wavelet transformation, using a 1-Kbp window-averaged melting profile over a wide range of scales from 20 Kbps to 5 Mbps, at steps of 20 Kbps. Subsequently, the scale-averaged wavelet power spectra were computed for examining the underlying rhythm of fluctuations in power over various scales.

We also randomly chose some melting map stretches of 2~3 Mbp in length from various chromosomes, and performed the wavelet analysis within each nonoverlapping 10-Kbp or 20-Kbp segments. This analysis was done at base-pair level, that is, using scales from 2 bp to 1,024 bp at steps of 2 bp to capture oscillations at a high resolution.

## Results

### Macroscopic Features of the Human Genomic Melting Map

The fundamental feature of a melting map is the occurrence of thermodynamically stable and unstable regions, having relatively high and low melting temperatures, respectively. For the human chromosomes, we obtained statistics of melting map features using nonoverlapping averaging window sizes from 10 bp to 1 Mbp. Table 1 shows the basic statistics of melting temperatures averaged over nonoverlapping 1-Kbp segments. The highest 1-Kbp window averaged melting temperatures varied between the chromosomes in the range 86.35 °C to 88.40 °C, where the latter occurred within the 60,074th 1-Kbp segment of Chromosome 20, containing a number of GC-rich repeat motifs such as (CGG)_n_. The lowest melting temperature per chromosome varied from 48.85 °C to 51.82 °C, where Chromosome 16 displayed the low 48.85 °C at the 10,501th 1-Kbp segment, which fully overlapped with a repeat of (TA)_n_ type. The 100-bp segments displayed similar statistics (see http://meltmap.uio.no/results/misc/meltmap_chr_global_stat_100bp.pdf). The broad ranges of melting temperatures in the human melting map were in contrast with those of the randomized chromosomes, which had melting temperatures in a narrow range of 70 ± 6 °C. A similar picture arose for the GC contents of 1-Kbp windows. In the human genome, the bulk of GC contents (in 1-Kbp nonoverlapping window) ranges roughly from 20% to 80%, while in the randomized chromosomes, the ranges are about six times as narrow.

**Table 1:**
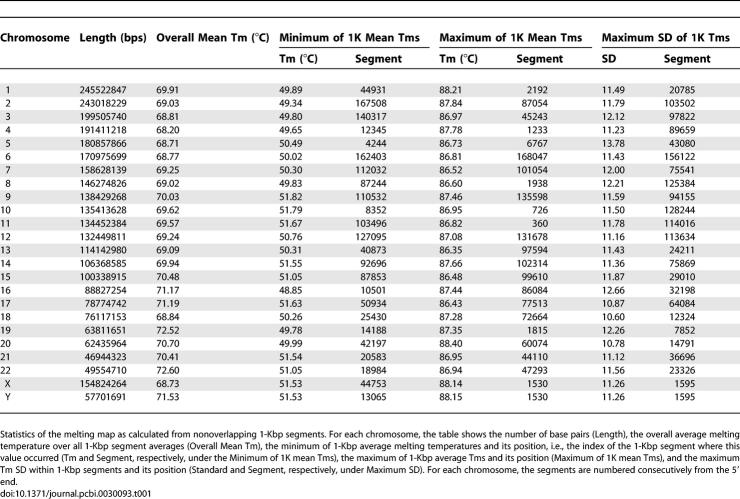
Overall Satistics of the Human Genomic Melting Map

We compared the correlations between GC content and the melting temperature using different window sizes, ranging from 10 bps to 1 Mbps. The human genomic melting map showed a very strong correlation with the local GC content within windows of 1 Kbp and larger; above 0.99 for all chromosomes (see Figure 2). As also shown in Figure 2, the correlation was found to be relatively low at small window sizes, increasing roughly log-linearly until reaching more than 0.98 at a window size of 500 bps. Thus, a likely interpretation is that the main features differentiating the GC content and melting temperature lies in the sub-500 bp range. Figure 2 also shows that, for all window sizes, the Tm and GC correlation was smaller for the randomized chromosomes than for the human chromosomes.

**Figure 2:**
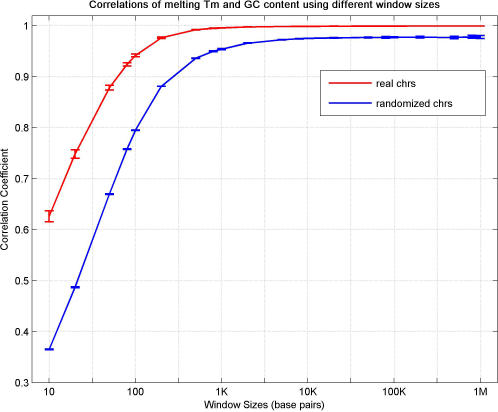
Correlations of Melting Temperature (Tm) with G + C Content The correlation coefficients between GC content and Tm are plotted as a function of window sizes. For each chromosome, excluding the segments which contain unknown bases (N's), the correlation coefficient was calculated from all pairs of GC content and average Tm over all nonoverlapping segments of a given window size. Across the chromosomes, the average correlation coefficients and SDs were calculated for each window size. The figure shows the average correlations with SDs (error bars) for window sizes from 10 bp to 1 Mbp for the human chromosomes (red) and the randomized chromosomes (blue).

### Correlation to Biological Features

Correlating a genome-wide recombination rate [38] with the melting map, we observed ten chromosomes where the melting temperature and recombination rates (at a 1-Mbp resolution level, in a gender-mixed population) correlated positively with a significant *p*-value of below 4.16E-4 (using Bonferroni correction), as shown in Table 2. Chromosomes 4 (r = 0.602), 5 (r = 0.599), and 13 (r = 0.560) displayed the highest correlations. For three chromosomes (15, 20, and 21), there was a negative correlation. Between the SD of the melting temperature over averaged 1-Mbp segments and the recombination rate, the correlation was weaker.

**Table 2:**
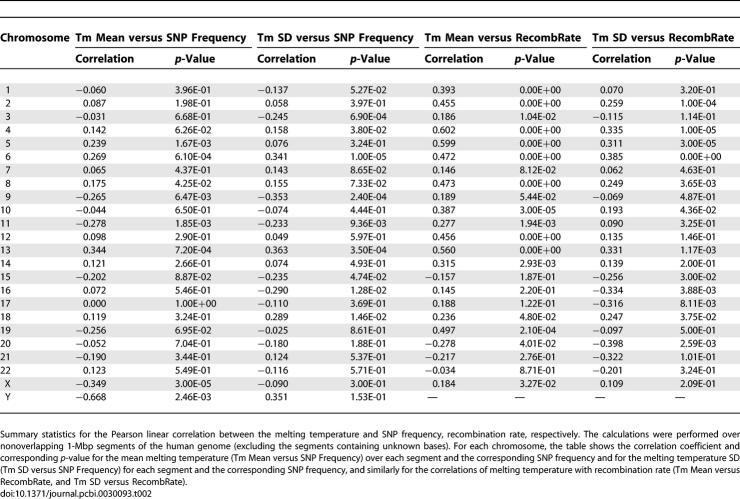
Correlations between Melting Temperature and SNP Frequency, Recombination Rate

Similarly, the Pearson correlations of the SNP frequency distribution [45] with the mean and SD of the melting temperatures were calculated for all chromosomes using 1 Mbp nonoverlapping windows. No strong correlations between these features were found to be statistically significant (see Table 2). SNP frequencies in *flat* segments on Chromosome 21 were investigated but did not reveal strong correlation with melting temperature (unpublished data).

### Analysis of Regions with High and Low Melting Temperatures

For a more detailed analysis of the stable and unstable regions, we defined the melting temperatures above 90 °C and below 50 °C as extreme temperatures (see http://meltmap.uio.no/results/extreme_tms.html). We defined stable (i.e., high-Tm) and unstable (i.e., low-Tm) regions as consecutive stretches of extreme high/low temperatures. Twenty high-Tm regions (average melting temperature 90.25 °C, average region length 347 bps) and 20 low-Tm regions (average melting temperature 49.5 °C, average region length 460 bp) were randomly chosen from various chromosomes. EpiGRAPH analysis (see Methods) between these stable and unstable regions (see http://meltmap.uio.no/results/EpiGraph/061011_125400_423443447_Attributes.html) indicated that unstable regions were associated with AT richness, low levels of evolutionary conservation, and high SNP frequency, and also exhibited frequent overlap with tandem repeats. The stable regions correlated with not only physical parameters of DNA, such as high solvent accessibility (as illustrated in Figure 3A), high DNA rise and roll, but also informational content, such as gene overrepresentation. By comparing the melting temperature distributions of exonic and nonexonic regions of each chromosome, we further observed that for most of the chromosomes, there seemed to be an offset of about 2 °C toward higher temperature for exons, compared with nonexons (see http://meltmap.uio.no/results/exon_vs_nonexon.html).

**Figure 3:**
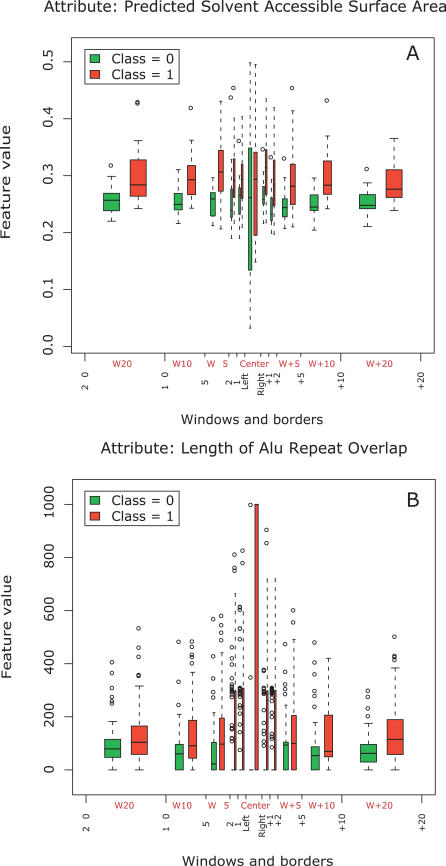
EpiGRAPH-Generated Diagrams Comparing Genomic Regions with Distinct Melting Profiles Displays boxplots comparing two genomic features between regions of high and low melting temperature (A) and *flat* and *nonflat* melting segments (B). Standard boxplots are drawn for the region itself and for ten windows surrounding the region, from −20 Kbp to +20 Kbp *(x*-axis), in order to capture neighborhood effects. The *y*-axis shows averages and distribution of the analyzed genomic feature. For each window, two boxplots are drawn, one for each class of melting profiles. (A) Regions are characterized by the extreme melting temperatures observed throughout the human genome. “Class 0” comprises 20 regions having low melting temperatures (below 50 °C in all cases), while “class 1” comprises 20 cases having high melting temperature (above 90 °C in all cases). Comparison with the average solvent-accessible surface area of the DNA (as predicted for each base pair using sequence trimers for which solvent accessibility has been established experimentally by the hydroxyl radical method [64]) shows that regions of high melting temperature exhibit substantially higher values than regions of low melting temperature. This is true not only for the region itself (center boxplot), but to a lesser extent also for its sequence neighborhood. (B) Regions are characterized by a *flat*/*nonflat* segmentation algorithm of the melting profile. “Class 0” contains 50 flat segments having an end-to-end step height of ±0.11 °C or less, while “class 1” contains 50 nonflat segments defined as having an end-to-end step height of ±6 °C or more. All segments were taken from Chromosome 21, exhibit an equal melting temperature of 68 °C and a segment length of 19 or 20 bps. Comparison with the average length of Alu repeat overlap per 1,000 base pairs (as identified by RepeatMasker) shows that flat regions are typically free of Alu repeats, while nonflat regions frequently exhibit substantial overlap with Alu repeats.

### Melting Domain Segmentations

The perhaps most interesting aspect of a melting map may in fact lie within the melting domain segmentation. In a macroscopic view, the human genomic melting map broadly follows the local GC content. However, with increased resolution, the cooperative melting characteristics appear as expected. Relatively flat plateaus of nearly equal melting temperatures are widespread, interspersed with step-like areas of both minor and large changes. We applied our segmentation definitions to examine the details of the melting map and its cooperativity. We thus performed an EpiGRAPH comparison of the annotation differences between two classes of randomly selected cases among the *up*/*down* versus *flat* segments of lengths 20~22 bp from Chromosome 21, all having average Tm = 68 °C. The *up*/*down* class consisted of 50 segments with end-to-end step heights above ±6 °C. The *flat* class consisted of 50 flat segments with end-to-end step heights below 0.1 °C (see http://meltmap.uio.no/results/EpiGraph/061119_190322_497232509_Attributes.html). A statistically significant association between Alu-type SINE repeat structures and the *up*/*down* class was found, compared with the *flat* class (see Figure 3B). There were also an increased number of transcription start sites and genes in the *up*/*down* segments compared with the *flat* segments. The analysis was repeated with similar findings using Chromosome 22. These observations applied also to the segmented data of nonoverlapping 100-bp windows based on SDs of the melting temperatures (see http://meltmap.uio.no/results/EpiGraph/061109_194104_300525259_Attributes.html). When comparing segments with increasing melting temperatures (*up* segments) with decreasing temperatures (*down* segments) (in a 5′-3′ orientation), no significant differences were found.

In an attempt to capture possible functional differences between *flat* segments of different sizes (below 100 bps), we performed EpiGRAPH analyses using 25 cases from each of two classes, which contained *flat* segments at ~68 °C in the length of ~60 bps and ~20 bps, respectively, from Chromosome 21. Both options of choosing data with and without repeat masking were examined. It was found that the correlation of the two classes with the kurtosis and SD of the physical parameters (twist, rise, and roll), as well as those for C and G frequency, had Bonferroni-adjusted *p*-values being equal or smaller than 10^−6^, but no significant correlation to functional annotations were found (unpublished data).

To further investigate the relationship between sequence statistics and melting features, we examined the GC contents of all 50 bp *flat* segments of Chromosome 21. Intriguingly, we observed not one, but three, distinct bands in a scatter plot (see Figure 4), indicating that regions having identical sequence composition (i.e., equal GC content), may have qualitatively different structural stabilities (i.e., melting temperatures). We extended the analysis also to *flat* segments of lengths 20, 100, 150, and 200 bp. The separation between the three bands increases toward approximately 5 °C with decreasing segment length and, on the other hand, it disappears at approximately 200 bp. This phenomenon can also be observed for other human chromosomes, as well as for the randomized chromosomes. We found a simple rule that relates the three bands to neighboring regions and cooperativity. We grouped all the 50 bp *flat* segments into three categories (I, II and III) based on the difference between the average melting temperatures of the segments and their neighbor regions (also 50-bp long) at both sides. The segments in category I had higher melting temperatures on both sides than on themselves, and those in category III had lower melting temperatures on both sides than on themselves. The other segments were clustered into category II. When Figure 4 was color-coded according to this category definition, we found that the three visually distinct bands overlapped very well with the three mathematically defined categories. This provides an understanding of the impact on DNA thermal stability from neighboring regions in terms of cooperativity [15], and this could not have been revealed by sequence-based statistics alone.

**Figure 4:**
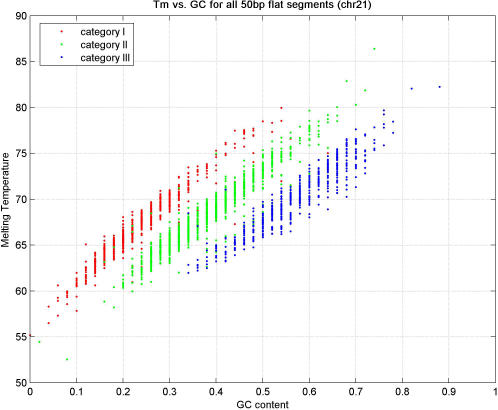
Scatter Plot of Melting Temperature versus GC Content of Flat Melting Segments Using Chromosome 21, the relationship between local GC content and melting temperature was examined for all flat segments of 50 bps. Figure 4 shows the scatter plot of melting temperature versus GC content. Each data point in this figure represents a 50-bp flat segment. The red dots represent those segments that have higher melting temperatures (Tm) in its neighboring regions at both sides (denoted as category I). The blue dots represent those that have lower Tm in its neighbors (denoted as category III). And, the green dots represent those that have lower Tm in one side neighbor and higher Tm in another (denoted as category II).

### Periodic Patterns in the Genomic Melting Map

Wavelet analysis on the low resolution melting map (i.e., using averaged Tm of nonoverlapping 1-Kbp windows across the genome) was performed in order to uncover possible oscillating patterns of melting temperatures along the whole sequence. Although different chromosomes did not reveal an identical pattern of periodicities, ~200 Kbp, ~400 Kbp, and ~1 Mbp were the common wavelengths observed in multiple chromosomes. This was also conducted for the GC content (using 1-Kbp nonoverlapping windows), and it tended to fall into the same general picture as that of the melting map. In contrast, no visible peaks could be found in the spectral analysis of the randomized chromosomes (see http://meltmap.uio.no/results/wavelet_global.html).

As it was possible that some periodic patterns with scales fewer than 1,000 bps also exist locally, we performed high-resolution (scales from 2 bps to 1,024 bps) spectral analysis on several randomly chosen ENCODE regions (see http://meltmap.uio.no/results/wavelet_local.html). In these analyses, a frequency of approximately 150 bps, roughly corresponding to the nucleosome length, was frequently seen. We also observed a general oscillation pattern in the range of 500~600 bps in most of the regions examined. A sample plot of local wavelet analysis is shown in Figure 5.

**Figure 5:**
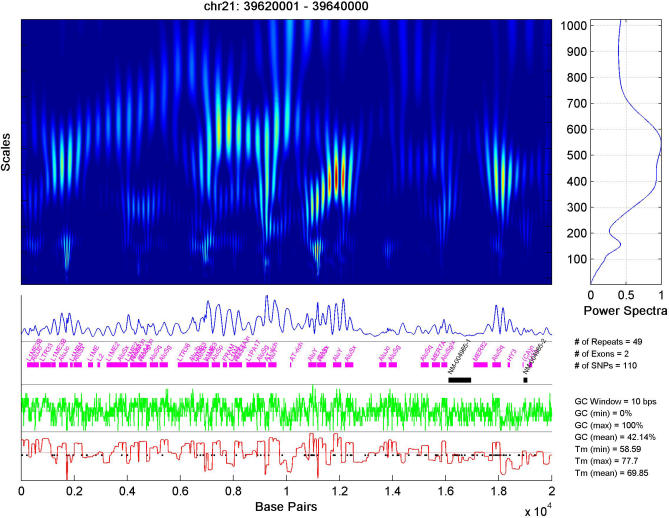
Local Spectral Analysis Local spectral analysis was performed on several randomly chosen 2-bp segments from various chromosomes. The segments were further divided into subsegments of 20 Kbp for which spectral analysis was performed individually. Wavelengths in the range of 2 bp to 1,024 bp, in steps of 2 bp, were assessed for each segment. This figure shows a representative segment (chr21: 39,620,001–39,640,000). The main part of the figure shows a heat map representation of the results from the spectral analysis. Below the main part, the power spectrum along the bases of the segment is displayed, followed by annotations of repeat structures and exons residing in the given segment. Below this again, the GC content profile (based on 10-p nonoverlapping windows), and then the melting map (as extracted from the corresponding region of the genomic melting map), are shown. The black dots in the melting curve represent the locations where known SNPs occur. On the right hand side, the power spectrum over the wavelengths is displayed; normalized to a value of 1 for the maximum.

## Discussion

We here present the complete melting map of the human genome. While we find a high correlation (0.99) between the local GC content and the melting map averaged in 1-Kbp windows for all human chromosomes, Figure 2 demonstrates that when calculations are performed on windows of fewer than 500 base pairs, the correlation is much smaller. This suggests that the characteristics of cooperativity being present in the melting map (as steps and flat plateaus) are not observable from GC content, as expected. For instance, at the window size of 100 bps, only 88.8% of the variation in the melting map could be attributed to the changes of GC content, while the rest should be attributed to the cooperativity. This is a qualitative difference that apparently correlation based on larger than 500 bp windows fails to recognize.

The randomized chromosomes derived from the selected randomization procedure were intended to serve as a baseline for comparison and for verification of our analyses. As these were required only to have the same overall frequencies of As, Cs, Gs, and Ts as the corresponding human chromosomes, the di-nucleotide composition was not preserved in the randomization procedure. Other randomization procedures would be worthwhile exploring for biological questions. The randomized chromosomes displayed a much more uniform GC content than the human chromosomes. As a consequence, the mosaic structure of the human chromosomes, presumably reflecting biological function, was not preserved. The correlations for the human chromosomes between DNA annotations and melting map features were not found for the randomized ones, well in line with our expectations.

We correlated the melting map with various physical and functional features of the genome at several levels of resolution in a first exploration of the information contained. The recombination rate [30,39] and the SNP frequency [46,47] have previously been reported to be positively correlated to the GC content. In a recent study on compositional symmetry of DNA and recombination rate, a negative correlation was found, indicating that asymmetry favors recombination [48]. As asymmetry will reflect regions of changing melting temperature, this was expected. In an examination of the SNP frequencies in flat melting segments, we only observed relatively minor variations in the extreme ends of the temperature range. For the remainder of the temperature range of flat segments, there appears to be a constant frequency of SNPs. Thus, more detailed studies are required to clarify whether DNA bubble openings may be important for SNP-inducing processes. Also, the chromosomally variable correlation of recombination and the melting map remains to be elucidated. Recently, an isochore map was published [49], that relies on observing the SD of the GC content in 100-Kbp windows, and defining the borders of isochores as abrupt shifts. It is not unlikely that the domain nature of the DNA melting map could be helpful, for defining isochores by melting temperatures, or for defining borders of GC isochores, although these topics remain to be explored.

In a zoomed-in view, the domain structure of the melting map is distinct from the GC content. To explore possible correlations between these domains and the plethora of other existing annotations for the human genome, it was necessary to extract the corresponding segments from the melting map in an automated way. No clear and rigorous definition of a melting domain exists, and it is not known how accurately domains can be determined from a melting map. Thus, we tried several approaches to locate segments at various resolution levels. Given a constant size of a window, we defined a *flat* window as having minimal SD of melting temperature, as opposed to *nonflat* windows that have high SD. To identify melting segments of various lengths, we chose an approach based on incremental step of consecutive melting temperatures. Both approaches were shown to be useful for this first exploratory effort.

Future studies, however, should address the segmentation aspect more elaborately. Better segmentation algorithms can be applied, for example, by modifying existing algorithms used for analyzing the GC contents [33,34]. A better knowledge of how the melting cooperativity manifests itself in a melting map may also contribute to the development of segmentation algorithms. In an approach of Yeramian et al. [50], the thermodynamic boundaries are located from a set of probability profiles at a range of temperatures, instead of using the melting map, but an automated method has not been provided. Another strategy is to calculate the bubble boundary locations directly, instead of first calculating the probability profiles and the melting map, followed by a segmentation to extract the information. For example, an algorithm for calculating the possible locations of melted and helical regions (stitch profiles) has been developed [51,52], but as its computation time scales quadratically, a genome-wide analysis must await the development of a linear version of that algorithm.

However, using the simple segmentation algorithm described, we were able to identify, and to some extent quantify, the effect of cooperativity between neighboring regions. We clearly demonstrated that the influence of neighbor regions is visible for the segments at lengths fewer than 150 base pairs, and increases in importance down to 20-bp segments. Also, it is observed that the cooperative effect depends on the length of the central segment, melting temperature differences between neighbor regions, and the lengths of neighboring regions. Cooperativity is generally not considered for most computer prediction algorithms of biological functions. As has been shown by Benham et al*.* for promoter locations in prokaryotes [53], it seems reasonable to expect improved predictions taking this aspect into consideration.

The extreme instances of high and low melting temperatures were statistically compared with existing annotations. The low-Tm regions naturally coincided with AT-rich regions, but were also found to coincide with regions of poorer conservation and relatively frequent SNP and repeat occurrence. The high-Tm regions correspondingly were found to be associated with GC-rich sequences, but more interestingly also to various physical parameters of DNA, such as high solvent accessibility and rise, and a higher association to genes. These findings are generally expected from the underlying sequence composition. It is for instance well-known that the gene frequency is higher in GC-rich regions, as are the bendability and B to Z conformation transitions. It is thought that these parameters relate to a propensity for gene transcription [54].

As the cooperative effect was found to be more pronounced for shorter melting segments, we focused on those of length fewer than 100 bps. Among these, a selection of *flat* and *nonflat* segments significantly identified Alu type repeats as a major contributor of *nonflat* segments. The general structure for these retrotransposed sequences of a few hundred base pairs consists of a GC-rich transcription start site, a variable middle part, and an AT-rich tail part [55]. In fact, we show that the Alu-type repeats represent a considerable fraction of the areas in the genome having steep melting temperature changes.

An overrepresentation of transcription start sites and increased gene frequency was also found in nonflat segments, as compared with flat segments. Recently, it has been observed that Alu-type sequence may have significant effects on gene expression, either through their influence on alternative splicing, through adenosine-inosine editing, or through protein translation influences [56]. Alu sequences have also been reported as having an increased fraction of SNPs [57], both in the GC-rich body, but also in the GC-poor tail of the Alu sequences, possibly related to increased recombination rates, thus underlining the possibility that AT replication slippage may be significant in the generation of SNPs in the AT-rich tail of the Alu sequence, as suggested previously [57].

We found that exonic sequences had a shift toward higher melting temperature, compared with non-exons across the whole spectrum of melting temperatures, as well as a larger tail at the high melting temperature side. Others have previously found correlations between the occurrence of exons and relatively stable, high-Tm regions [58,59]. However, this seems to be a species-dependent feature, possibly due to the occurrence of long intron regions in higher eukaryotes. In a study of genes where the introns had been removed, a correlation between thermodynamic boundaries and exon boundaries was suggested [60], and explained as a propensity of intronic sequences to be inserted at the boundary between open and closed DNA. This correlation also conforms well with the correlation between the GC content and exons, as previously described [61].

Previously, others have performed spectral analysis of selected regions of the human genome GC content and intra-strand asymmetry [42,62], in order to uncover wavelengths or oscillations along the DNA, and reported two significant periods (110 Kbp and 400 Kbp), which roughly correspond to sizes relevant for DNA loops and vertebrate replicons. We could identify these features in the genomic melting map as well. The computational cost for a genome-wide spectral analysis at base pair level resolution is presently too high. However, in a local wavelet analysis of several ENCODE selected genomic regions, we could identify a wavelength of approximately 150 bps. Some supportive studies for this observation also can be found [63–66], for instance showing that there is a general correlation between nucleosome complex local DNA double helix curvature and DNA chain bending as a function of the sequence composition.

This paper is primarily concerned with establishing basic relationships between the human genomic melting map and available biological annotations. We are aware that the melting model employed in this work was developed for in vitro predictions. DNA in vivo, being much more densely packed together with histones and other macromolecules, seems too encumbered to form melting bubbles freely. Nevertheless, single-stranded regions are widespread in vivo, driven by molecular motors, rather than by temperature. Both replication and transcription rely on local opening of the DNA to take place, and also these processes should be scrutinized for possible dependencies on the melting map. For instance, DNA mutation rates may be related to the probabilities and lifetimes of bubbles exposing the bases. In a study using the Dauxois–Peyrard-Bishop model on selected transcriptional promoters, it was indicated that the algorithm could identify the transcription start site [67], although the validity of this finding is disputed [68]. Studies using the SIDD model have shown that sites susceptible to opening correlate with replication origins and transcriptionally active regions [69–71]. Such topics should be addressed at the genomic level. The observation that neighboring segments, or potential bubbles, influence each other suggests that any prediction algorithm of sequence features related to single-stranded states would benefit greatly from including cooperativity as represented by the melting map. Today, such algorithms, for example, for transcription factor binding sites prediction (see recent review by Bajic et al. [72]), are often hampered by large numbers of false positive predictions. We expect that prediction algorithms relying on sequence motifs alone could be improved.

The central hypothesis for this melting map exploration is that the predictions of in vitro melting may reflect also the in vivo behavior. It is reasonable to believe that the sequence-dependent bubble openings are functionally important in vivo. However, the correlations with biological annotations do not necessarily indicate causalities, and their possible physical or biological origins cannot be deduced from this preliminary work alone. Some correlations may stem from sequence composition due to evolutionary changes, reflected in the melting map, but with no relation to bubble openings and functional role. Further studies of the melting map and its association to annotations and DNA structures are clearly warranted and at present made possible on a genomic scale. One such interesting topic would be the evolutionary aspects of the melting curve across related species with available sequence information. It is our opinion that the development of further refined melting models, that include more aspects of the in vivo physical constraints and flexibility of chromatin DNA as actually experienced in the different settings in the nucleus, could benefit from the knowledge gained in studies like the present one. This would significantly influence the understanding of the mechanisms behind a number of central structural and functional aspects of cells.

The following materials (including online tools, downloadable data, and results) can be accessed via the Web page http://meltmap.uio.no/: 1) brief introduction of the project (http://meltmap.uio.no/intro.html), 2) source code of the melting map calculation (http://meltmap.uio.no/code.html), 3) online tool for calculating parameters of loop entropy factor approximation (http://meltmap.uio.no/tools/loopentropy.html), 4) human genomic melting map data files (based on UCSC release *hg17*) (http://meltmap.uio.no/rawdata.html), 5) browser of human genomic melting map (http://meltmap.uio.no/browser), 6) human genomic melting map being included in the Ensembl genome browser as a DAS source (http://meltmap.uio.no/tools/ensembl.html), and supplementary analytical results (http://meltmap.uio.no/analysis.html).

## Abbreviations

PFF: Poland–Fixman–Freire
SD: standard deviation
